# Immune regulation and prognostic prediction model establishment and validation of PSMB6 in lung adenocarcinoma

**DOI:** 10.3389/fgene.2024.1458047

**Published:** 2024-10-23

**Authors:** Haiyang Zhao, Kexin Luo, Meihan Liu, Yuanze Cai, Siman Liu, Shijuan Li, Yongsheng Zhao, Hongpan Zhang

**Affiliations:** ^1^ Affiliated Hospital of North Sichuan Medical College, Nanchong, Sichuan, China; ^2^ North Sichuan Medical College, Nanchong, China; ^3^ Department of Thoracic Surgery, Affiliated Hospital of North Sichuan Medical College, Nanchong, Sichuan, China; ^4^ North Sichuan Medical College, Innovation Centre for Science and Technology, Nanchong, China; ^5^ Department of Oncology, Affiliated Hospital of North Sichuan Medical College, Nanchong, China; ^6^ Nanchong Central Hospital, Nanchong, Sichuan, China; ^7^ Therapeutic Proteins Key Laboratory of Sichuan Province, Nanchong, China

**Keywords:** lung adenocarcinoma, PSMB6, immunotherapy, immune infiltration, tumor immune microenvironment

## Abstract

Lung cancer is one of the most common malignant tumors, and patients are often diagnosed at an advanced stage, posing a substantial risk to human health, so it is crucial to establish a model to forecast the prognosis of patients with lung cancer. Recent research has indicated that proteasome 20S subunit 6 (PSMB6) may be closely associated with anti-apoptotic pathways, and proliferation transduction signals in tumor cells of different tumors. However, the precise role of PSMB6 in the immunoregulatory processes within lung adenocarcinoma (LUAD) is yet to be elucidated. By analyzing the TCGA database, we discovered a positive correlation between the expression of PSMB6 and tumor growth trends, and lung adenocarcinoma patients with elevated PSMB6 expression levels had a worse prognosis. Our findings suggest a close correlation between PSMB6 expression levels, immune cell infiltration and immune checkpoint gene expression, which suggests that PSMB6 may become a new independent prognostic indicator. In addition, we developed a prognostic model of PSMB6-regulated immune infiltration-associated genes by analyzing the link between PSMB6 and the immune microenvironment. This model can not only predict the prognosis of lung adenocarcinoma but also forecasts the patient’s reaction to immunotherapy. The validity of this research outcome has been confirmed by the GSE31210 and IMvigor210 cohorts. Analysis of the Kaplan-Meier Plotter database indicates that individuals with elevated levels of PSMB6 expression exhibit a poorer prognosis. Additionally, *in vitro* experiments demonstrated that knockdown of PSMB6 inhibits the proliferation, migration, and invasion of lung adenocarcinoma cells while promoting their apoptosis. Overall, our findings suggest that PSMB6 could remarkably influence the management and treatment of lung adenocarcinoma, opening new avenues for targeted immunotherapeutic strategies.

## 1 Introduction

Globally, lung cancer ranks as one of the most common and lethal malignant tumors, with its annual incidence and mortality rates continuing to rise. According to the International Agency for Research on Cancer (IARC), the overall 5-year survival rate for lung cancer patients remains alarmingly low at only 17.7% (Bray et al., 2018; Tang et al., 2022; Lahiri et al., 2023). Among the various subtypes, lung adenocarcinoma (LUAD) has emerged as the predominant form, accounting for nearly 60% of new lung cancer cases (Lu et al., 2019). Despite the employment of multiple anti-cancer strategies including surgery, chemotherapy, and radiotherapy, there remains a critical need for more effective treatment approaches to manage or cure lung adenocarcinoma (Yasumoto et al., 2009). Despite recent advancements in the field that have resulted in the development of immunotherapy mediated by checkpoint suppression, this approach still has notable limitations. Specifically, it benefits only a minority of patients, approximately 20%–30%, and issues related to toxicity accumulation and drug resistance continue to persist (Tang et al., 2022). Therefore, there is a pressing need to explore reliable models for predicting the prognosis of lung adenocarcinoma, which could significantly aid clinicians in making more informed clinical decisions.

The proteasome, a complex responsible for the post-ubiquitination proteasomal degradation of misfolded or typically short-lived intracellular proteins, which constitutes over 80% of all intracellular proteins, plays a crucial role in this context (Guo et al., 2022; Chauhan et al., 2004). Studies have already demonstrated that cancer cells exploit the proteasome system to facilitate their abnormal proliferation, evade apoptosis, and degrade tumor suppressor proteins to aid their proliferation and metastasis (Shen et al., 2013; Chauhan et al., 2004). Moreover, the expression levels of proteasome genes, such as PSMB6, have been observed to increase in many types of cancers, suggesting that PSMB6 could potentially serve as a molecular therapeutic target (Guo et al., 2022; Bruzzoni-Giovanelli et al., 2015; Ding et al., 2020). However, the role of the proteasome β-subunit PSMB6 gene in LUAD has not been fully revealed, especially its interaction with immune infiltration.

Based on these findings, our study employed detailed bioinformatics analysis and *in vitro* experiments. Our research indicates that high expression of PSMB6 is associated with poor immune infiltration and prognosis, while knocking down PSMB6 promotes apoptosis in lung adenocarcinoma cells and inhibits their proliferation, metastasis, and invasion. Furthermore, we developed a highly reliable clinical prognostic prediction model. This model not only enhances our understanding of the role of PSMB6 in the immune microenvironment but also aims to assist clinicians in formulating personalized medical strategies to ultimately improve the prognosis of patients with this challenging disease.

## 2 Material and methods

### 2.1 Data collection and processing

Data from The Cancer Genome Atlas (TCGA) database was collected, including clinical information and PSMB6 expression patterns, encompassing 33 distinct tumor types. Access to data was facilitated via UCSC Xena (Lee et al., 2019) (https://xena.ucsc.edu/) and analysis was performed utilizing R software, specifically employing packages suited for genomic data analysis (https://www.R-project.org). The research focuses on extracting somatic mutation information from the TCGA database and building a model, while the Gene Expression Omnibus (GEO) dataset GSE31210 (Peng et al., 2017) was employed to validate the prognostic capabilities of our model. The efficacy of immunotherapy drugs is verified by the IMvigor210 cohort of atezolizumab in the treatment of urothelial cancer (Mariathasan et al., 2018). Based on the Kaplan-Meier Plotter database (Hou et al., 2017) (https://kmplot.com/analysis/), we analyzed the prognosis of PSMB6 in lung adenocarcinoma patients and plotted Kaplan-Meier survival curves.

### 2.2 The correlation between PSMB6 and pan-cancer

To investigate the correlation between PSMB6 expression and its clinical implications across various cancers, we utilized R software. We conducted univariate Cox regression analyses using the “survival” package to evaluate the prognostic significance of PSMB6 expression levels across 33 different cancer types. Hazard ratios (HR) less than 1 indicated that higher PSMB6 expression correlates with a lower risk of mortality. The analyses were adjusted for potential confounders, including gender, age, and tumor stage, to ensure robustness in our findings. Further, we evaluated the correlation between PSMB6 expression and various clinical parameters such as clinical stage, grading, and TNM staging. We considered a *p*-value threshold below 0.05 to determine statistical significance in our study.

### 2.3 Cell culture and transfection

A549 and H1299 lung adenocarcinoma cells and BEAS-2B human normal lung epithelial cells were placed in 10% DMEM (Gibco; Thermo Fisher Scientific, Inc.) and cultured in a humidified incubator at 37°C with 5% CO₂.

PSMB6 silencing was achieved in A549 and H1299 cells by transfection with PSMB6 siRNA (GenePharma, China). After 24–48 h of transfection, the medium was removed for cellular behavioral analysis. The PSMB6-specific siRNA oligonucleotides were synthesized according to the following target sequences:

siPSMB6#1: sense strand sequence 5′-AUU​CGC​CGU​UGC​CAC​UUU​ATT-3′, antisense strand sequence 5′-UAA​AGU​GGC​AAC​GGC​GAA​UTT-3’; siPSMB6#2: sense strand sequence 5′-ACU​GGG​AAA​GCC​GAG​AAG​UTT-3′, antisense strand sequence 5′-ACU​UCU​CGG​CUU​UCC​CAG​UTT-3’.

### 2.4 RNA extraction and quantitative RT-qPCR

Total RNA was extracted from clinical samples using the standard TRIzol protocol (Thermo Fisher Scientific, United States). Complementary DNA (cDNA) was synthesized from 1 μg of RNA using the PrimeScript™ FAST RT Reagent Kit with gDNA Eraser (TAKARA Bio Inc., Shiga, Japan). RT-qPCR was performed using the TB Green^®^ Premix Ex Taq™ II (TAKARA Bio Inc., Shiga, Japan) on the BIO-RAD CFX Connect Real-Time System (serial number: 788BR06671, Bio-Rad Laboratories, Inc., Hercules, CA, United States). Relative RNA expression levels were quantified using the 2^ΔΔCt method, with GAPDH serving as the endogenous reference gene. At least two independent experiments were conducted, with each experiment including at least three technical replicates. Analyses were performed using the Bio-Rad CFX96 Maestro Manager 2.0 software. Data analysis and graphical representations were conducted using Prism 10 software. Statistical significance was determined using unpaired t-tests. The primers for PSMB6 are as follows: Forward Primer: GAC​ACC​TAT​TCA​CGA​CCG​CAT​T, Reverse Primer: TAA​AGA​GGC​TGG​CTG​CTG​TGT. The primers for GAPDH are as follows: Forward Primer: CTT​TGG​TAT​CGT​GGA​AGG​ACT​C, Reverse Primer: GTA​GAG​GCA​GGG​ATG​ATG​TTC​T.

### 2.5 Western blot assay

The harvested cells were placed on ice, and after discarding the culture medium, they were washed three times with pre-cold PBS. Then, an appropriate amount of RIPA lysis buffer (Epizyme, China) was added, along with phosphatase inhibitors and protease inhibitors at a ratio of 100:1. The cells were scraped off using a cell scraper and lysed in an ultrasonic lysis solution for 1 min, followed by centrifugation at 12,000 g for 15 min to collect the supernatant. The protein concentration was determined using the BCA assay (Epizyme) after incubating for 30 min, measuring absorbance at 562 nm. The protein samples were diluted with 5× loading buffer (Epizyme) at a ratio of 4:1 and heated in a metal bath for 10 min to denature the proteins. A 10% Western blot gel was prepared using Epizyme’s one-step method, and the electrophoresis was run at 80 V for 30 min and then at 120 V for 60 min. For transfer, a PVDF membrane was used under the conditions of 250 mA for 60 min. After that, blocking was performed in a rapid blocking solution for 20–30 min, followed by washing three times with TBST. The primary antibody (PSMB6, Proteintech) was incubated overnight, and the next day a secondary antibody (Proteintech) was used for incubation followed by detection using Epizyme’s ultra-sensitive ECL luminescent solution and a Bio-Rad imaging instrument. Data processing was conducted using ImageJ, with all primary antibody dilutions at 1:3000 and secondary antibody dilutions at 1:5000. The E-cadherin and N-Cadherin antibodies were provided by Affinity Biosciences (China), while BAX and BCL-2 primary antibodies were provided by Huabio (China).

### 2.6 CCK8 assay

The Cell Counting Kit-8 (Beyotime, China) was used to assess cell proliferation. After resuspending the cells, they were counted and diluted, and 100 μL of medium containing 5,000 cells was added to each well. After transfection, 10 μL of CCK-8 reagent was added to each well at 0 h, 24 h, 48 h, and 72 h, and the absorbance was measured at 450 nm 1 hour later. Each group had five replicate wells, and the experiment was repeated three times to ensure result reliability.

### 2.7 Transwell migration assay

The cells were starved for 24 h, followed by digestion and resuspension. A total of 40,000 cells and 200 μL of serum-free DMEM were added to the upper chamber of each Transwell, while 600 μL of DMEM containing 10% fetal bovine serum was added to the lower chamber. The upper chamber was carefully immersed in the lower chamber liquid using sterile tweezers, and the 24-well plate containing the Transwell inserts (Corning, United States) was incubated at 37°C for 24 h.

For the invasion assay, Matrigel (Beyotime, China) was required. According to the instructions, Matrigel was diluted at a ratio of 1:8 and coated in the upper chamber. After 24 h, the liquid in the upper chamber was removed, and the wells were washed three times with 600 μL PBS, followed by fixation with formaldehyde. Staining was performed using crystal violet, and the samples were then observed and photographed under an electron microscope. Cell counting was conducted using ImageJ software. Each experiment was repeated three times to ensure the reliability of the results.

### 2.8 Wound healing assay

Logarithmically growing cells were seeded in a six-well plate at a density of 1 × 10^5^ cells per well, with three replicate wells for each group. Once the cells adhered and formed a monolayer, a 200 μL pipette tip was used to create a vertical scratch in each well, ensuring the scratch remained straight. The wells were washed with PBS to remove any floating cells, and the remaining cells were placed in a 37°C, 5% CO₂ incubator to be cultured in low serum medium (1%). Images were taken under a microscope at 0, 24, and 48 h post-scratch. Each experiment was repeated three times. Finally, ImageJ software was used to measure the distance of cell migration and calculate the migration rate.

### 2.9 Flow cytometric analysis

Cells were collected using trypsin without EDTA, followed by two washes with PBS to collect 1–5 × 10^5^ cells. These cells were then added to 500 µL of Binding Buffer and gently pipetted to form a single-cell suspension. Next, 5 µL of Annexin V-APC (keyGEN, China) was added and mixed, followed by the addition of 5 µL of PI (keyGEN) and mixed again. The reaction mixture was incubated in the dark at room temperature for 10 min. Detection was performed using a flow cytometer, with the excitation wavelength for APC set at Ex = 633 nm and emission wavelength at Em = 660 nm, detecting red fluorescence using the FL4 channel; for PI, the excitation wavelength was Ex = 488 nm and emission wavelength Em ≥ 630 nm, detected through the FL3 channel.

### 2.10 Analysis of immune cell infiltration and immune characteristics

We evaluated the proportional distribution of 22 distinct immune cell populations in lung adenocarcinoma using the CIBERSORT method and analyzed the association of these immune cells with PSMB6 expression (Newman et al., 2015). Using the ESTIMATE algorithm, we compared the difference between the PSMB6 high and low expression groups in terms of stromal scores, immune scores, and ESTIMATE scores. Furthermore, we employed Spearman Correlation Analysis to further demonstrate the association of these scores with PSMB6 expression (Yoshihara et al., 2013). Finally, we utilized the MCPcounter (Becht et al., 2016a) method to classify immune cell taxa in lung adenocarcinoma in more detail to understand the specific cell types present in the tumor microenvironment.

### 2.11 Genomic screening and gene set enrichment analysis

We performed GO enrichment analysis of genes associated with PSMB6 and immune infiltration by Spearman Correlation Analysis and screened for genes significantly associated with PSMB6 expression and immune scores (*p* < 0.001, correlation >0.3). We performed GO and KEGG analyses on the 367 genes screened using the clusterProfiler R package and then prioritized the top 10 most significant terms in each category for visualization. For pathway analysis, we used GSVA method to calculate pathway scores for the TCGA sample cohort. The Wilcoxon test was used to determine the difference in pathway activity between the PSMB6 high and low expression groups. Finally, we used single-sample genome enrichment analysis (ssGSEA) to analyze the correlation between these significantly different pathways.

### 2.12 Risk model construction

We screened 9 key genes with prognostic value by univariate Cox and Lasso regression analysis. Next, we calculated risk scores for each subject using multivariate Cox regression analysis and z-scored the risk scores. finally, the high and low risk groups were delineated with a cutoff of 0. An external dataset sourced from the Gene Expression Omnibus (GEO) was incorporated into our study for additional analysis, serving as an independent validation to assess the consistency and dependability of the established model. Comprehensive evaluation, including univariate and multivariate Cox analysis, Nomogram, and decision curve analysis.

### 2.13 Immunotherapy response and chemotherapy analysis

In this study, we analyzed an independent dataset of immunotherapy subjects to classify treatment outcomes into four categories: complete response (CR), partial response (PR), disease progression (PD), and stable disease (SD). Non-responders were classified as SD or PD, while responders were identified as belonging to the CR or PR category. To assess the difference in PSMB6 expression between responders and non-responders, the statistical Wilcoxon rank sum test was used to gain insight into the role of PSMB6 in immunotherapy efficacy.

### 2.14 Statistical analysis

In our study, all statistical analyses (including the calculation methods for *p*-values and confidence intervals) were performed using R language and Prism - GraphPad. The association between two groups of quantitative variables was estimated using the Pearson correlation coefficient, while intergroup comparisons were conducted using the *t*-test, analysis of variance (ANOVA), and rank-sum test. Data are presented as mean ± standard deviation (SD).

## 3 Result

### 3.1 The level of PSMB6 expression in pan-cancer and its prognostic value across cancer subtypes

**FIGURE 1:**
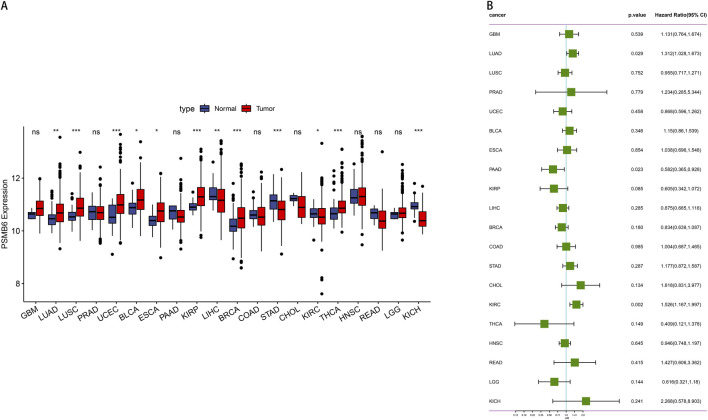
Expression and prognosis analysis of PSMB6 in pan-cancer. **(A)** Comparison of PSMB6 levels in normal and tumor tissues of different types of cancer. **(B)** Forest plot showing survival hazard ratios for various cancers based on PSMB6 expression.

We evaluated the pan-cancer expression profile of PSMB6 using data from the TCGA database. The analysis revealed a pronounced upregulation of PSMB6 expression in eight 8 kinds of tumors: Lung Adenocarcinoma (LUAD), Lung Squamous Cell Carcinoma (LUSC), Uterine Corpus Endometrial Carcinoma (UCEC), Bladder Cancer (BLCA), Esophageal Carcinoma (ESCA), Kidney Renal Papillary Cell Carcinoma (KIRP), Breast Cancer (BRCA), Thyroid Carcinoma (THCA). Conversely, a low expression of PSMB6 was observed in four specific types of tumors: Liver Hepatocellular Carcinoma (LIHC), Stomach Adenocarcinoma (STAD), Kidney Renal Clear Cell Carcinoma (KIRC), Kidney Chromophobe (KICH) (*p* < 0.05, Figure 1A). Furthermore, we performed a Cox survival analysis utilizing the PSMB6 expression as a basis. The findings demonstrated that elevated expression of PSMB6 is linked to decreased survival rates in LUAD and KIRC. However, in pancreatic adenocarcinoma (PAAD), elevated levels of PSMB6 expression correlate with a more favorable prognosis. (*p* < 0.05, 95% CI, Figure 1B).

### 3.2 Effect of PSMB6 expression on LUAD clinical outcomes

**FIGURE 2:**
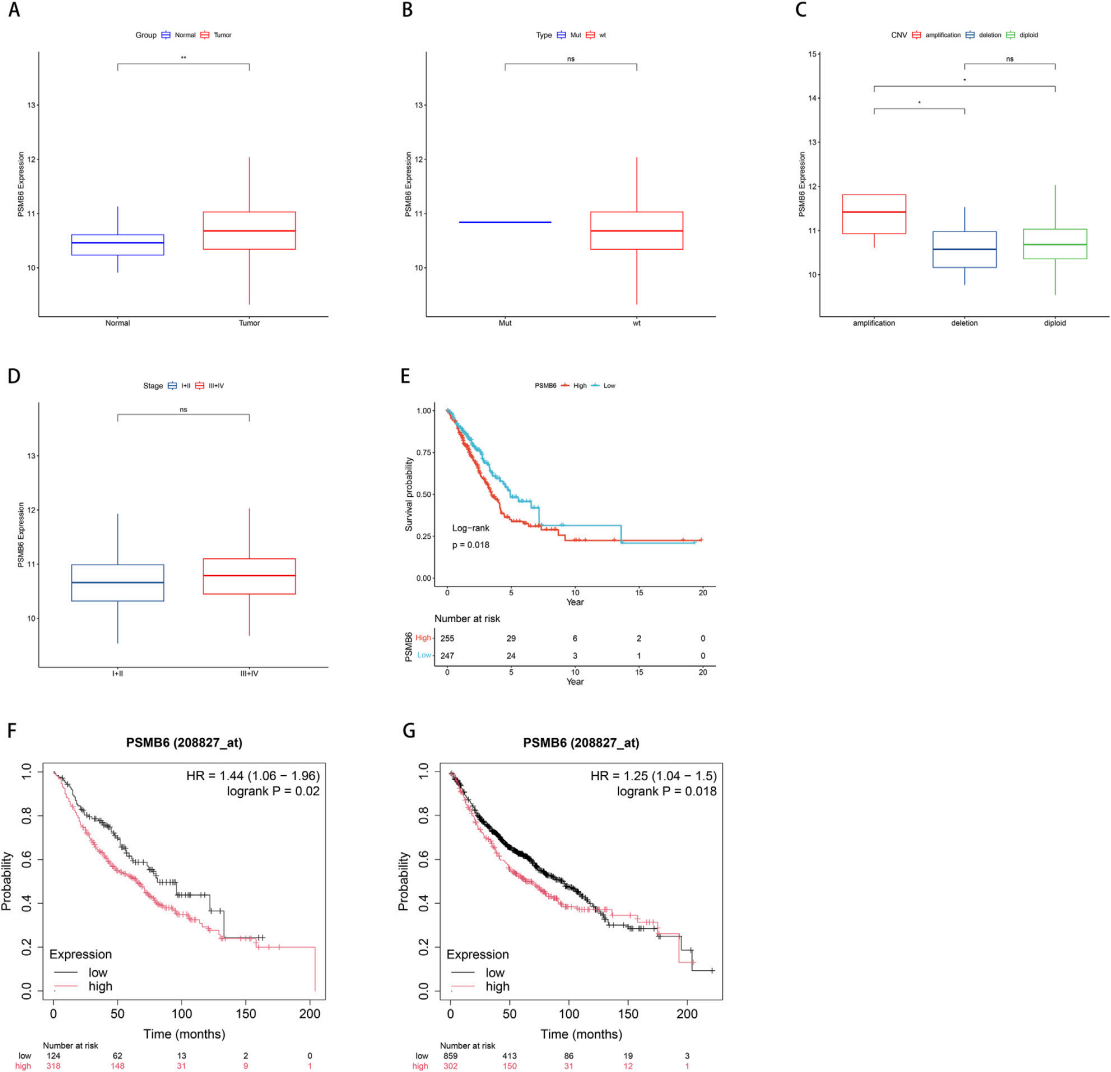
Correlation between PSMB6 and clinical characteristics. **(A)** The expression level of PSMB6 is significantly higher in tumor tissues compared to normal tissues. **(B)** There is no significant difference between the mutant gene and the wild-type gene in the expression level of PSMB6. **(C)** PSMB6 expression in samples with gene amplifications is significantly higher compared to those with deletions or diploids. **(D)** There is no significant differences in the expression level of PSMB6 between early (stages I-II) and advanced (stages III-IV) cancer stages. **(E–G)** K-M curve suggests that the prognosis of the low PSMB6 expression group is better.

We utilized data from the TCGA database to investigate the role of PSMB6 in lung adenocarcinoma (LUAD). This analysis focused on the differences in PSMB6 expression between normal and tumor tissues, as well as its relationships with single nucleotide variants (SNV), copy number variations (CNV), cancer stages, and patient survival. The results indicated that PSMB6 expression was significantly higher in tumor tissues compared to normal tissues (*p* < 0.01, Figure 2A). No significant differences in PSMB6 expression levels were observed when comparing the mutant group to the wild-type group (Figure 2B). In samples with gene amplification, PSMB6 expression was significantly higher than in samples with gene deletion or normal gene dosage (*p* < 0.05, Figure 2C). Comparisons between early-stage (I-II) and late-stage (III-IV) cancers showed no significant differences in PSMB6 expression levels (Figure 2D). Kaplan-Meier curve analysis revealed that high PSMB6 expression was significantly associated with lower patient survival rates compared to low expression patients (*p* = 0.018, Figure 2E). Additionally, we further performed an online prognostic analysis of PSMB6 in LUAD using the Kaplan-Meier Plotter website and plotted K-M curves. The findings indicated that patients with elevated PSMB6 expression had a poorer prognosis (*p* < 0.05, Figures 2F, G).

### 3.3 The correlation between PSMB6 expression and tumor microenvironment and immune infiltration

**FIGURE 3:**
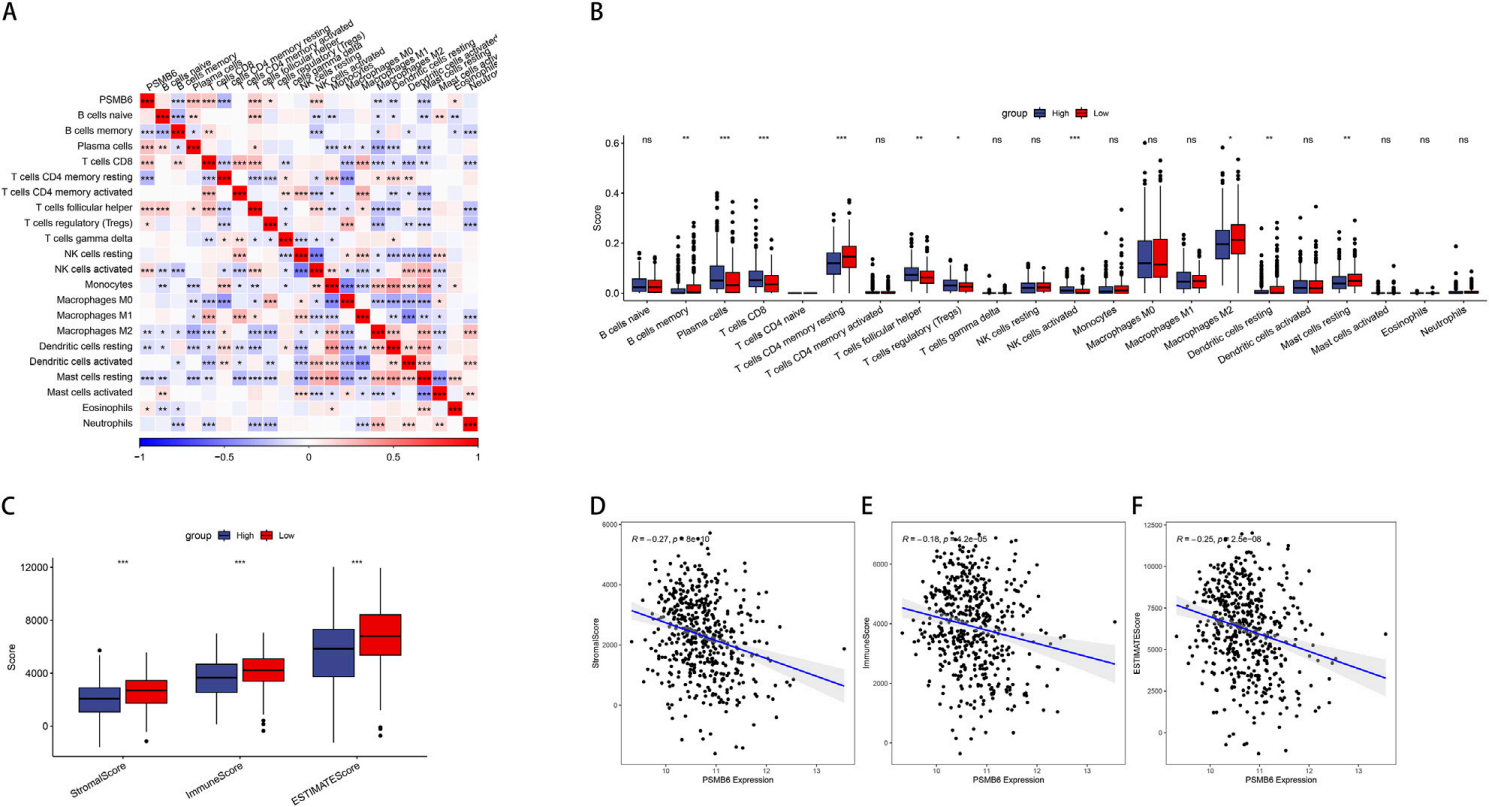
PSMB6 and immune cell infiltration. **(A, B)** The correlation of PSMB6 expression with immune cells score in LUAD. **(C)** The low PSMB6 group had higher stromal score, immune score and ESTIMATE score. **(D–F)** The expression of PSMB6 is negatively correlated with StromalScore, ImmuneScore and ESTIMATEScore.

To further compare immune cell scores between groups with high and low PSMB6 expression, we calculated the immune scores of 22 types of immune cells and used the median to divide high and low expression groups. Notably, the high expression of PSMB6 was positively correlated with the infiltration levels of plasma cells, CD8^+^ T cells, follicular helper T cells, regulatory T cells and activated NK T cells, while negatively correlated with the memory B cells, CD4 memory resting T cells, M2 macrophages, resting Dendritic cells and resting mast cells (*p* < 0.05, Figures 3A, B). Additionally, we investigated the relationship between the high and low PSMB6 expression groups in terms of stromal scores, immune scores, and ESTIMATE scores, and found significant differences (*p* < 0.001, Figure 3C). PSMB6 expression exhibited a marked inverse relationship with the stromal score, immune score, and ESTIMATE score (Figures 3D–F).

### 3.4 Correlation analysis of PSMB6 expression pathway

**FIGURE 4:**
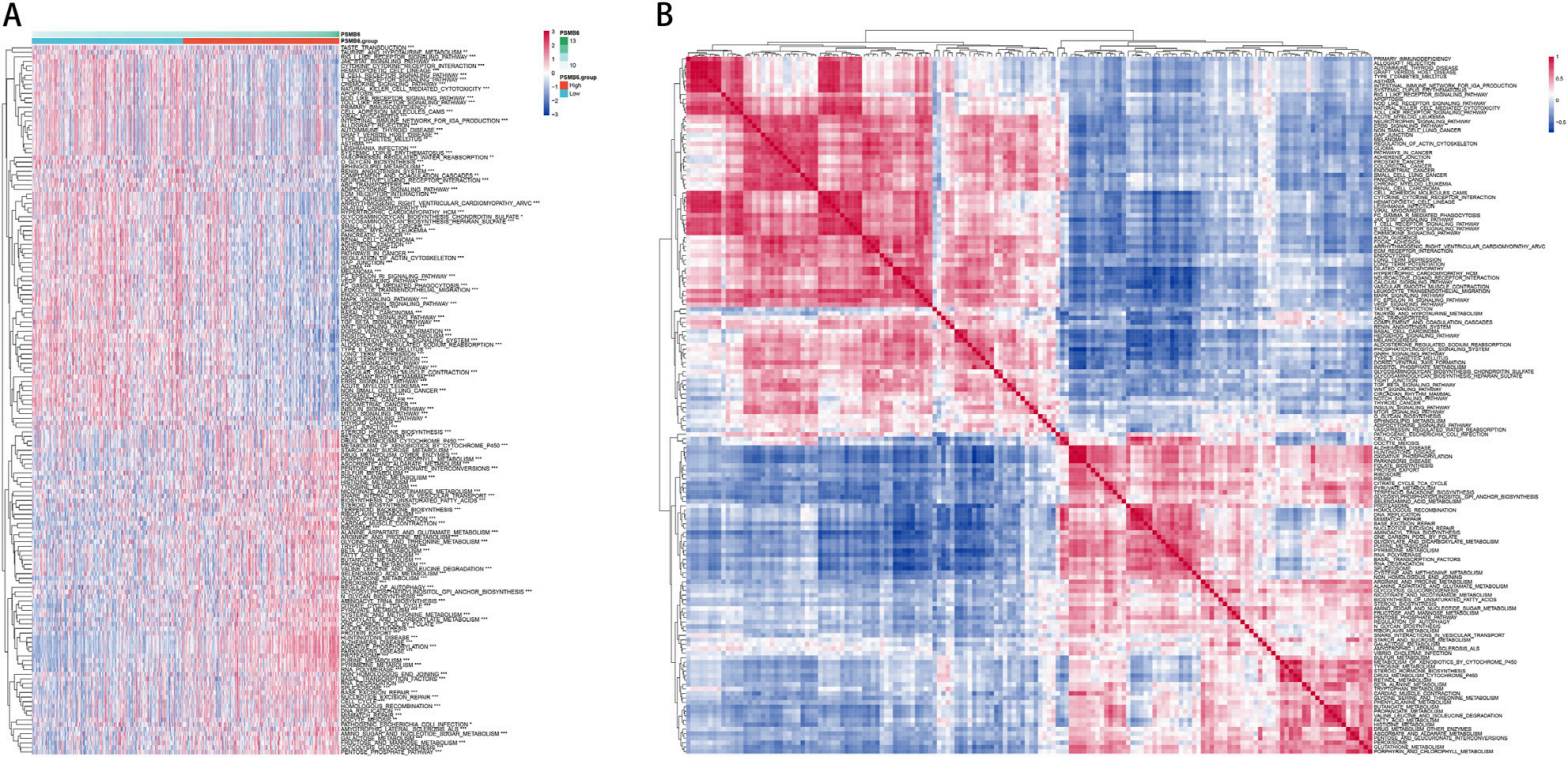
**(A, B)** PSMB6 high expression group and low expression group regulate different pathways.

To further explore the pathways potentially regulated by the PSMB6 gene, we used R software and the Gene Set Variation Analysis (GSVA) method to score pathways across individual TCGA samples based on their expression profiles. We then visualized these data using a heatmap, displaying the ssGSEA scores for each pathway and sample (Figure 4A). Simultaneously, we performed correlation analyses between these pathways to identify significant interactions and dependencies. Our analysis uncovered distinct associations between different pathways and the groups categorized by high and low levels of PSMB6 expression (Figure 4B).

### 3.5 A comprehensive analysis of the correlation and functional enrichment of PSMB6 with cancer immune signature genes

**FIGURE 5:**
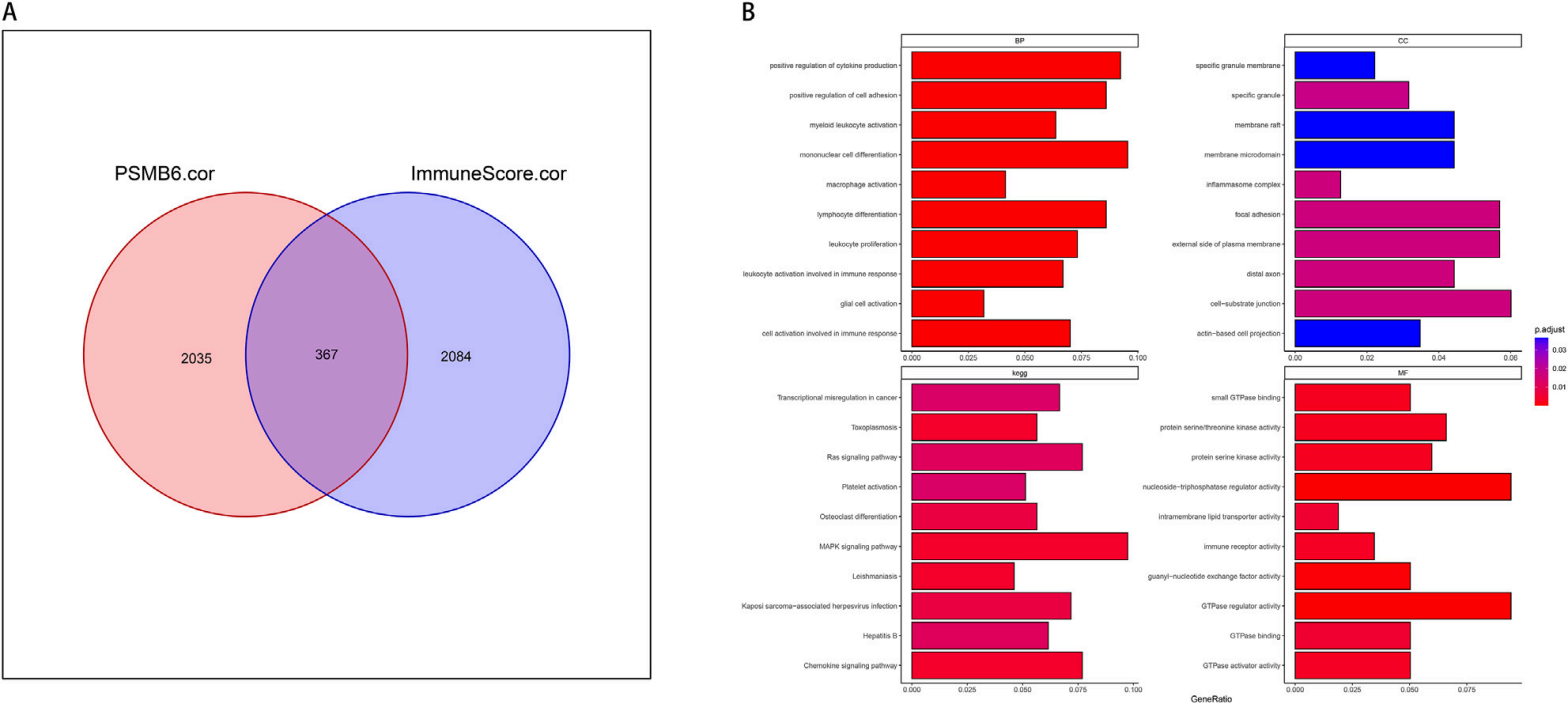
PSMB6 and immune signature genes and functional enrichment. **(A)** Venn diagram showing the intersection of 367 genes between 2035 PSMB6-related genes and 2084 immune score-related genes. **(B)** Barplot graph of the Gene Ontology Biological Processes, Cellular Components, and Molecular Functions enrichment analysis and Barplot graph of the Kyoto Encyclopedia of Genes and Genomes Pathways enrichment analysis.

We screened for genes associated with both PSMB6 and immune infiltration, identifying those with a correlation greater than 0.3 or less than −0.3. We took the intersection of all genes with PSMB6 correlation >0.3 or < -0.3 and immune score correlation >0.3 or < -0.3, and obtained 367 genes (Figure 5A). We then employed the clusterProfiler package to perform enrichment analysis on these 367 genes. We selected and visualized the top 10 most significant pathways in four categories: biological process (BP), cellular component (CC), molecular function (MF), and KEGG pathways. The GO analysis significantly enriched immune-related functional pathways, including positive regulation of cytokine production, leukocyte proliferation, and cellular activation involved in immune response. Concurrently, the KEGG analysis revealed notable enrichment in pathways such as the MAPK signaling pathway (Figure 5B).

### 3.6 Establish and validate the LUAD risk model

**FIGURE 6:**
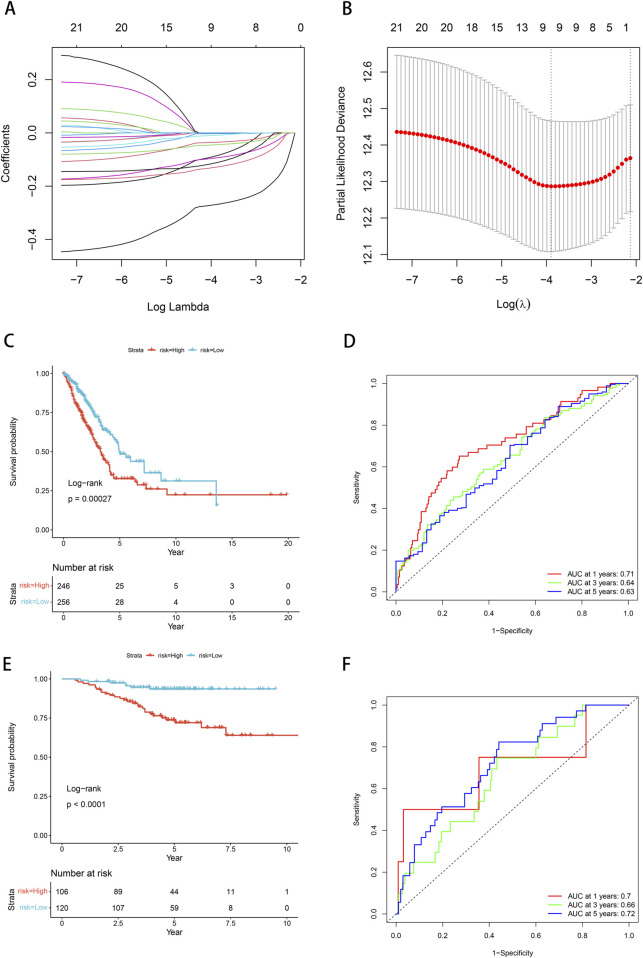
Prognostic model and validation. **(A)** Cross-validation of tuning parameter selection in LASSO models. **(B)** Risk model construction 9 identification of key genes. **(C)** The K-M curve of the TCGA cohort suggests that patients in the high-risk group have worse prognosis. **(D)** ROC curves and AUC for 1-, 3-, and 5-year OS in the TCGA cohort. **(E)** The KM curve validated by the GSE31210 data set suggests that the high-risk group has a worse prognosis. **(F)** ROC curves and AUC for 1-, 3-, and 5-year OS of GSE31210 data set.

We conducted univariate Cox proportional hazards regression analysis using expression profiling data from TCGA and associated gene and survival data. Subsequently, 21 genes were identified as having prognostic value and lasso regression was used to further compress these 21 genes, reducing the number of genes in the risk model (Figure 6A). Finally, we obtained 9 genes: ANKRD13A, CD200R1, GGA2, NLRP1, SLC18A2, XCR1, ZNF136, ZNF25, and ZNF441 (Figure 6B). We calculated risk scores using multivariate Cox analysis and then standardized these scores using z-score transformation.

Risk scores = “-0.13*ANKRD13A + -0.004*CD200R1+-0.295*GGA2+-0.042*NLRP1+-0.018*SLC18A2+-0.116*XCR1+-0.149*ZNF136+-0.154*ZNF25+-0.054*ZNF441”.

The Kaplan-Meier curve showed that low-risk patients had a better prognosis compared with high-risk patients (*p* = 0.00027, Figure 6C). Based on the ROC analysis, the model in the TCGA dataset demonstrated significant predictive value for LUAD patients, with an AUC of 0.71 at 1-year, 0.64 at 3-year, and 0.63 at 5-year (Figure 6D). We employed the GSE31210 dataset to substantiate the risk model proposed in this work, calculating the risk score for each sample based on its gene expression level, and plotting the distribution of these scores. The results obtained from the Kaplan-Meier curves were consistent with our model (*p* < 0.0001, Figure 6E). Based on the ROC analysis, high-risk patients exhibited a unfavorable prognosis, with an AUC of 0.7 at 1-year, 0.66 at 3-year, and 0.72 at 5-year (Figure 6F).

### 3.7 Association of risk models with clinicopathological features of LUAD

**FIGURE 7:**
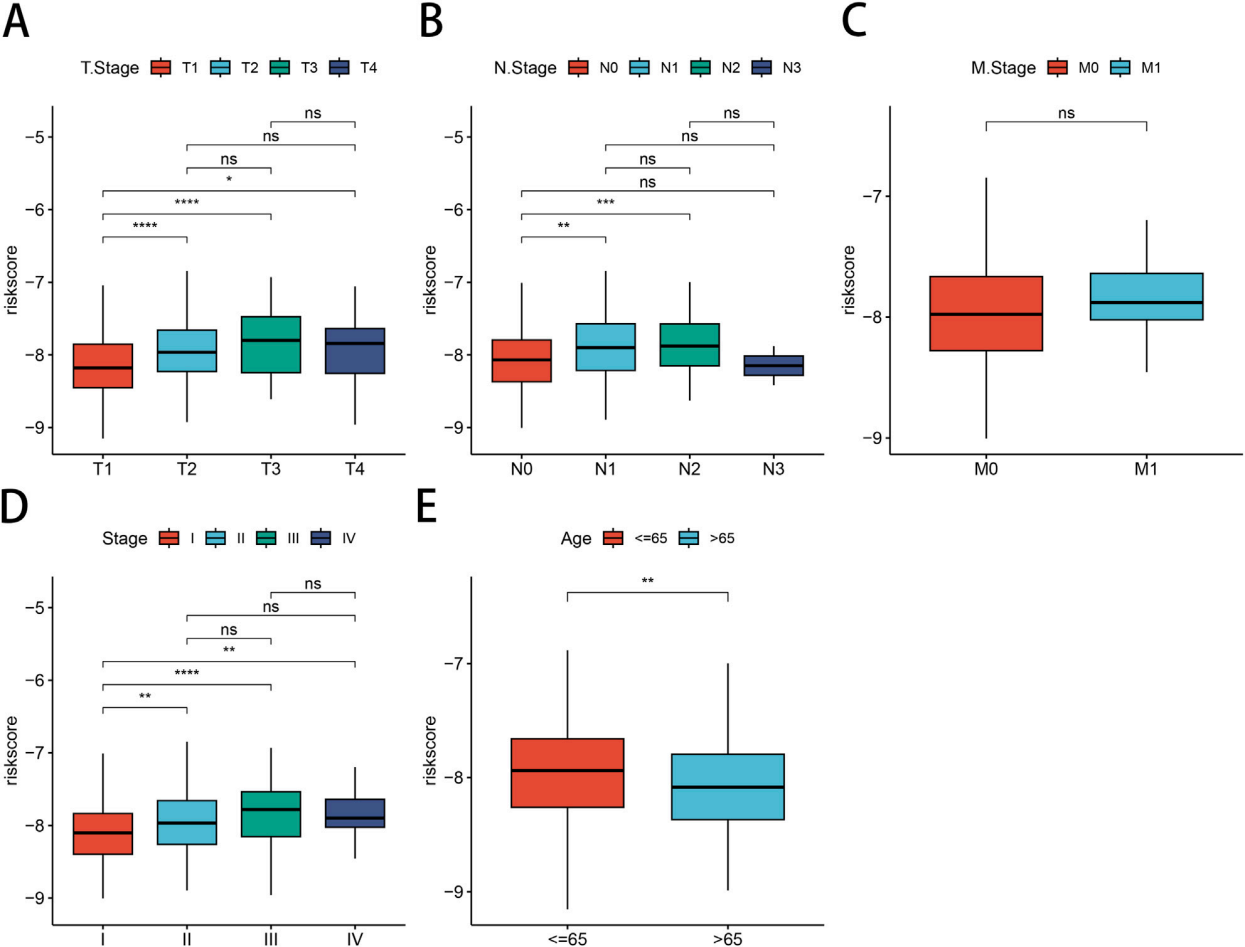
Correlation between risk models and clinical characteristics. Differential expression of risk models for LUAD patients according to clinical characteristics are presented: **(A)** T-stage, **(B)** N-stage, **(C)** M-stage, **(D)** clinical stage, **(E)** Age.

Next, we investigated the relationship between our risk modeling based on PSMB6 and clinical phenotypes in LUAD patients. We found that the median risk scores increased with tumor stage progression across (T) stages (Figure 7A), (N) stages (Figure 7B), and overall clinical stages (Figure 7D). This trend emphasizes the prognostic relevance of the PSMB6 risk score to cancer progression. However, our analysis did not detect a substantial disparity in risk scores among patients classified as stage M0 and those at stage M1 (Figure 7C). Interestingly, LUAD patients over 65 years of age presented lower risk scores (Figure 7E).

### 3.8 Analysis of association between LUAD risk score and immune cell infiltration

**FIGURE 8:**
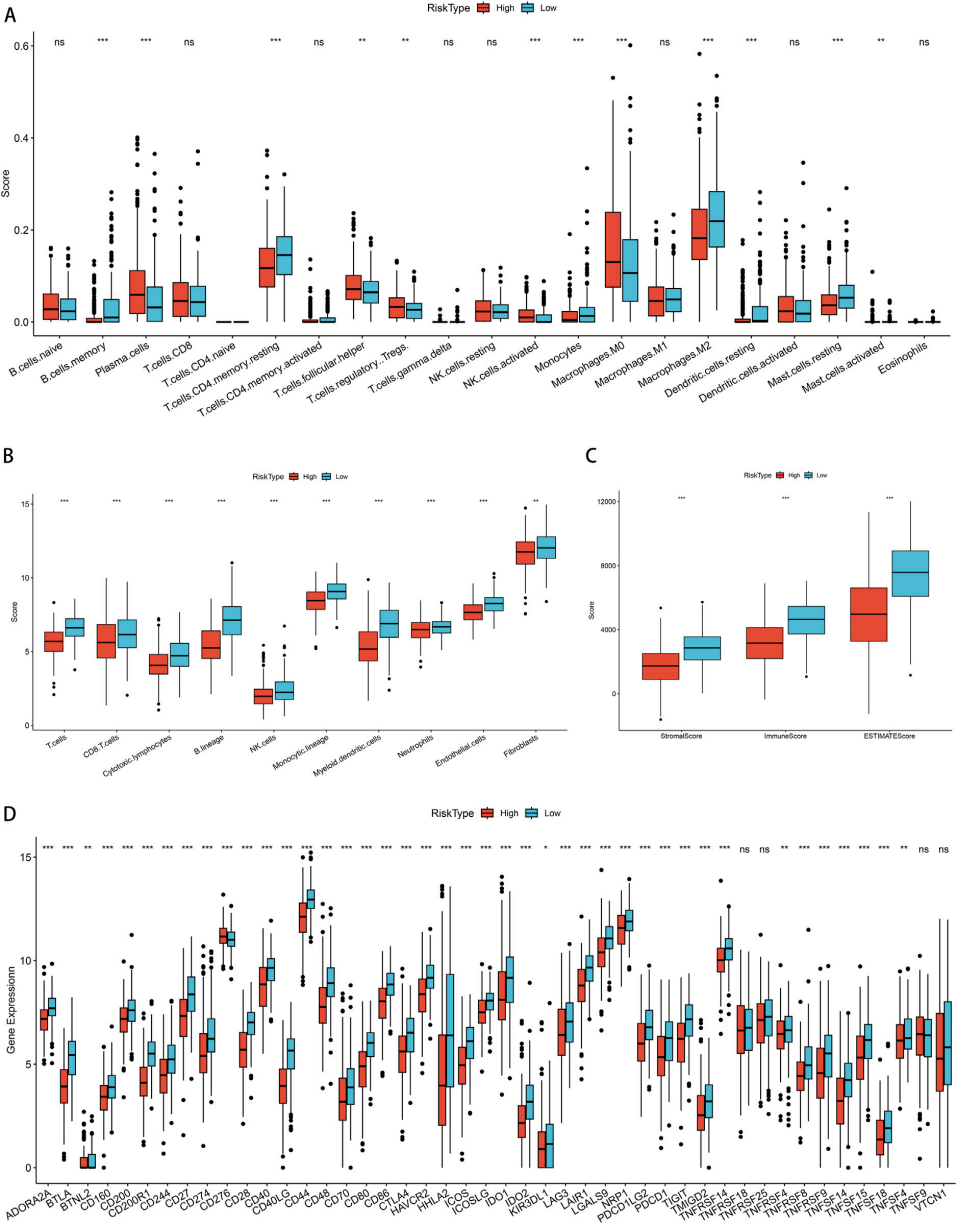
Correlation of immune cell infiltration among different PSMB6 risk groups. **(A)** Immune cell scores in different PSMB6 risk groups. **(B)** Immune cell scores in the high-risk group are lower. **(C)** StromalScore, ImmuneScore and ESTIMATEScore scores in the PSMB6 high-risk group are lower. **(D)** High expression of most immune checkpoints is positively correlated with PSMB6 low-risk group.

First, we conducted an examination to discern the variations in the levels of immune cell infiltration present within the groups categorized by low and high PSMB6 risk. We calculated the enrichment scores for various immune cell populations and identified substantial disparities in the extent of immune cell infiltration between the low-risk and high-risk groups. The results showed that the infiltration of memory B cells, CD4 memory resting T cells, monocytes, M2 macrophages, resting dendritic cells, and resting mast cells was more abundant in the low-risk group. In contrast, the high-risk group exhibited a more significant infiltration of plasma cells, follicular helper T cells, regulatory T cells, activated NK cells, and M0 macrophages (*p* < 0.01, Figure 8A). Next, we utilized the Microenvironment Cell Populations-counter (MCP-counter) method to assess the population abundance of tissue-infiltrating immune and stromal cell populations. We identified that the low-risk group exhibited notably elevated scores for T cells, CD8^+^ T cells, cytotoxic lymphocytes, B lineage cells, NK cells, monocytes, myeloid dendritic cells, neutrophils, endothelial cells, and fibroblasts (*p* < 0.01, Figure 8B). Furthermore, using the ESTIMATE method, we observed that the stromal score, immune score, and overall ESTIMATE score were all markedly diminished in the high-risk group as compared to the low-risk group. (*p* < 0.001, Figure 8C). Finally, in our analysis of immune checkpoint molecule expression between the low-risk and high-risk groups, we observed significantly higher expression of key immune checkpoint-related genes, including CD200R1, PDCD1, CD28, and CTLA4, in the low-risk group (*p* < 0.001, Figure 8D).

### 3.9 Validation of risk models using datasets from immunotherapy

**FIGURE 9:**
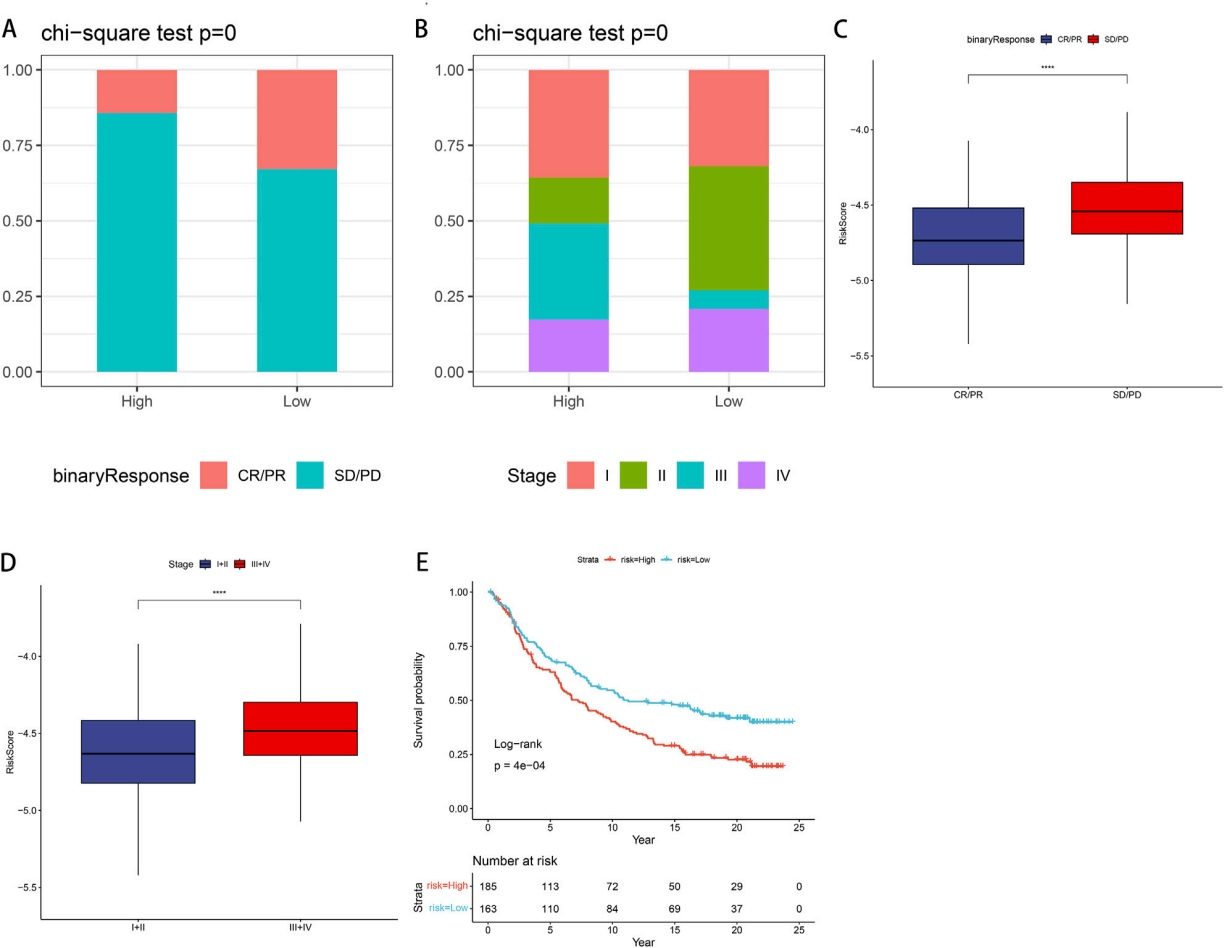
IMvigor210 data set validates immune efficacy and prognosis in LUAD patients **(A)** In the low-risk group, the proportion of non-responders to immunotherapy is lower. **(B)** In the low-risk group, the proportion of early-stage patients is higher. **(C)** Patients with SD/PD have higher risk scores compared with CR/PR. **(D)** Patients with stage III + IV have a higher risk score compared to stage I + II. **(E)** K-M curve shows that high-risk groups have poor prognosis.

We examined the efficacy of immunotherapy response in patients categorized into CR/PR and SD/PD, based on the risk stratification of PSMB6 into high-risk and low-risk groups. For external validation, we employed the IMvigor210 dataset to verify the robustness and reliability of our prognostic model. Our findings revealed a higher proportion of non-responders to immunotherapy within the high-risk group, as assessed in the treatment response evaluation (*p* < 0.05, Figure 9A), and a lower proportion of early-stage patients in the high-risk group (*p* < 0.05, Figure 9B). Moreover, patients in the SD/PD group exhibited significantly higher risk scores compared to those in the CR/PR group (*p* < 0.0001, Figure 9C). When comparing early (stages I + II) and late (stages III + IV) risk scores, we observed that earlier staging was associated with lower risk scores (*p* < 0.0001, Figure 9D). Finally, the K-M curve Finally, the K-M curve illustrated a notably poorer prognosis for patients classified within the high-risk category in contrast to those categorized within the low-risk group (*P* = 4e−04, Figure 9E).

### 3.10 Build and validate predictive nomograms

**FIGURE 10:**
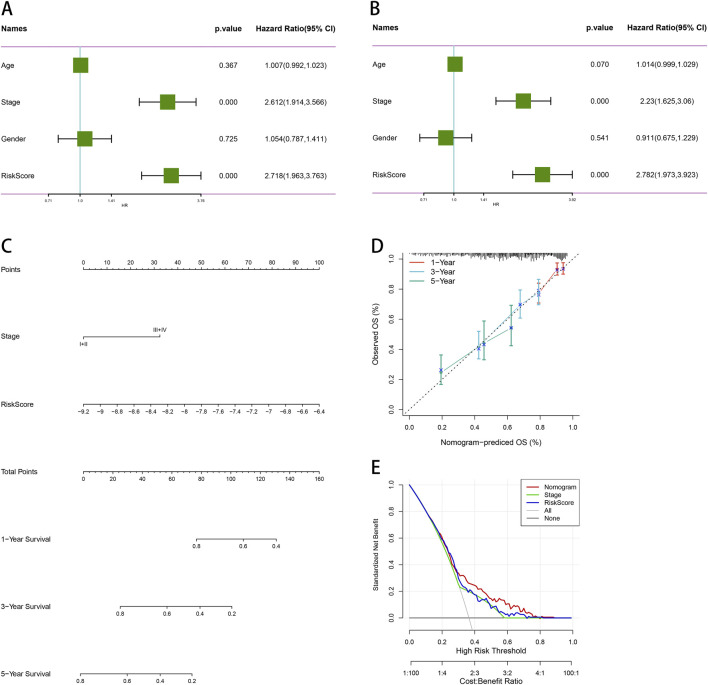
Comprehensive survival analysis and prognostic assessment of cancer patients. **(A)** Univariate regression analysis of age, stage, gender, and risk score. **(B)** Multivariate regression analysis of age, stage, gender and risk score. **(C)** Nomogram integrating PSMB6 with stage and gender as independent prognostic factors for LUAD. **(D)** Calibration curve of the nomogram. **(E)** The DCA curve suggests that Nomogram has the best prediction effect.

We conducted univariate and multivariate Cox regression analyses to evaluate other clinical variables, including age, sex, stage, and risk score, and to determine if our model operates independently of other clinical prognostic factors that may influence patient outcomes. Both stage and risk score were identified as independent predictors of overall survival (OS) (*p* < 0.05, 95%CI, Figures 10A, B). We then developed nomograms incorporating these independent prognostic biomarkers to estimate the one-, three-, and 5-year survival probabilities for individual patients (Figure 10C). The calibration curves for the nomograms showed that the predicted OS for 1 year, 3 years, and 5 years closely aligned with the observed OS (Figure 10D). Furthermore, we constructed a nomogram for predicting patient prognosis, which demonstrated high consistency with clinical predictions. DCA was used to compare the net benefit across different risk thresholds, indicating that the nomogram provided the most effective prediction (Figure 10E).

### 3.11 Knockdown of PSMB6 promotes apoptosis in LUAD cells and inhibits their proliferation

**FIGURE 11:**
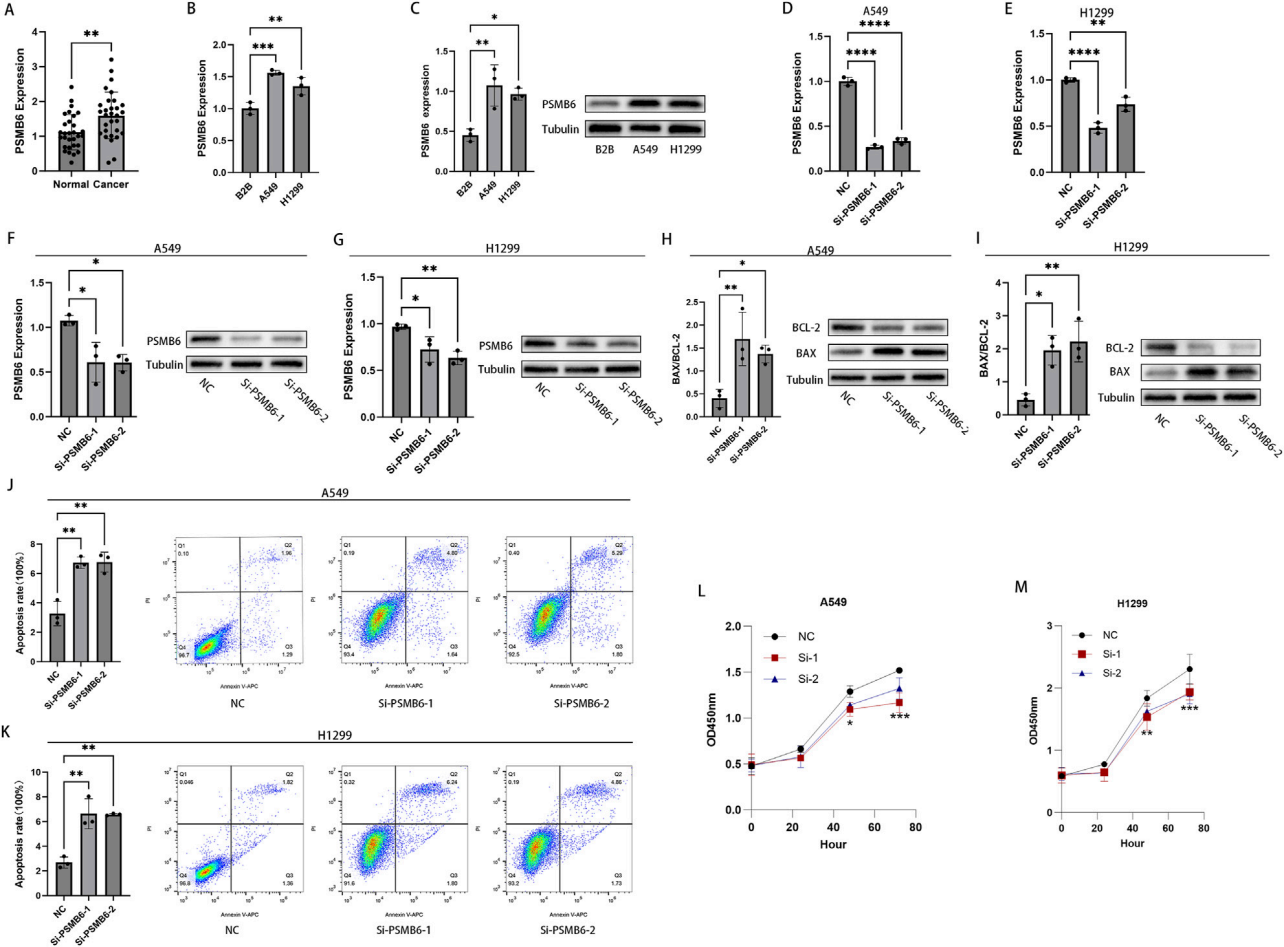
Knockdown of PSMB6 promotes apoptosis in LUAD cells and inhibits their proliferation. **(A, B)** The mRNA levels of PSMB6 were measured using RT-qPCR in 30 pairs of cancerous and normal tissues from LUAD patients, as well as in 3 cell lines: A549, H1299, and B2B. **(C)** WB analysis showed that the expression level of PSMB6 was significantly higher in A549 and H1299 cell lines compared to the B2B cell line. **(D–G)** Knockdown efficiency of PSMB6 in LUAD cells was verified by RT-qPCR and Western blot. **(H, I)** Western blot analysis was used to compare the expression levels of BCL-2 and BAX in PSMB6 knockdown A549 and H1299 cells with those in the control groups. **(J, K)** Apoptosis rates of A549 and H1299 cells with PSMB6 knockdown and their corresponding control cells without PSMB6 knockdown were measured using flow cytometry. **(L, M)** The cell growth rates of PSMB6 knockdown A549 and H1299 cells were determined using the CCK-8 method and compared with those of the control groups.

To validate the aforementioned findings, we performed RT-qPCR analysis on tumor samples from LUAD patients. The results showed that the expression of PSMB6 in 30 LUAD tumor samples was significantly higher than that in paired normal samples (*p* < 0.01, Figure 11A). Furthermore, both RT-qPCR and Western blot analyses indicated that the expression of PSMB6 was significantly elevated in the lung cancer cell lines A549 and H1299 compared to the normal lung epithelial cell line B2B (*p* < 0.05, Figures 11B, C). To further investigate the impact of PSMB6 on the apoptosis and proliferation of lung adenocarcinoma cells, we established PSMB6 knockdown A549 and H1299 cell lines through transient transfection (*p* < 0.01, Figures 11D–G). Western blot analysis revealed that in A549 and H1299 cells, knockdown of PSMB6 resulted in an upregulation of BAX expression and a downregulation of BCL-2 expression (*p* < 0.05, Figures 11H, I). Flow cytometry results indicated that the apoptosis rate of PSMB6 knockdown A549 and H1299 cells was significantly higher than that of the control group (*p* < 0.01, Figures 11J, K), further validating the role of PSMB6 in resisting apoptosis in lung adenocarcinoma cells. Additionally, CCK8 assays demonstrated that PSMB6 knockdown significantly inhibited the proliferation of A549 and H1299 cells (*p* < 0.05, Figures 11L, M).

### 3.12 Knockdown of PSMB6 inhibits the migration and invasion of LUAD cells

**FIGURE 12:**
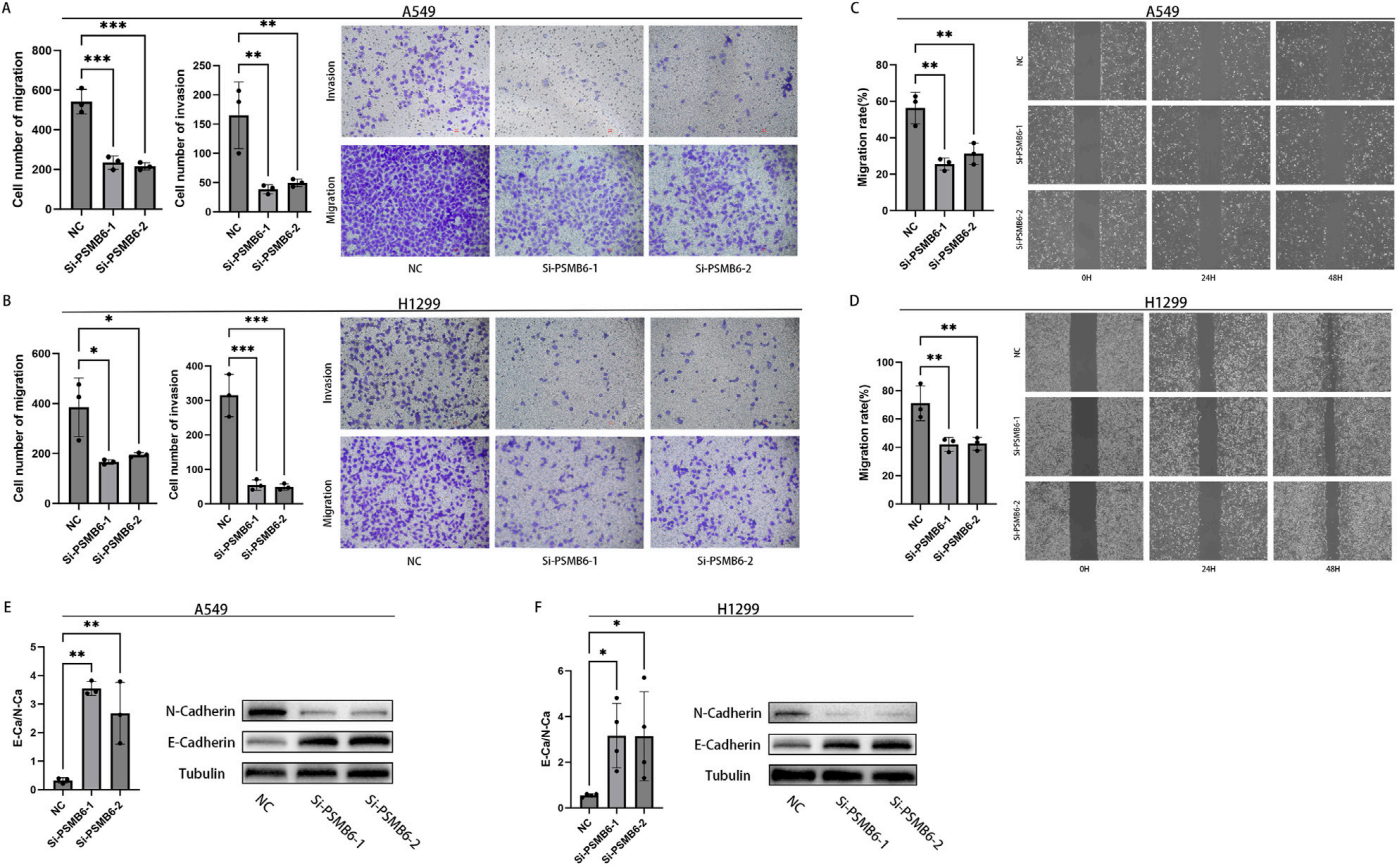
Knockdown of PSMB6 inhibits the migration and invasion of LUAD cells. **(A, B)** The migration and invasion abilities of A549 and H1299 cells with PSMB6 knockdown and their corresponding control cells without PSMB6 knockdown were assessed using a Transwell migration assay. **(C, D)** The migratory ability of A549 and H1299 cells with PSMB6 knockdown and their corresponding control cells without PSMB6 knockdown was assessed using a wound healing assay. **(E, F)** Western blot analysis was used to compare the expression levels of E-Cadherin and N-Cadherin in PSMB6 knockdown A549 and H1299 cells with those in the control groups.

We next explored the effect of PSMB6 on the migration and invasion of lung adenocarcinoma cells. The Transwell migration assay demonstrated that the migration and invasion capabilities of A549 and H1299 cells with PSMB6 knockdown were significantly lower than those of the control group (*p* < 0.05, Figures 12A, B). The wound healing assay indicated that knocking down PSMB6 expression in A549 and H1299 cells led to a marked decrease in their migration abilities, consistent with the results from the Transwell migration assay (*p* < 0.01, Figures 12C, D). This further confirms the promoting role of PSMB6 in the metastasis of lung adenocarcinoma cells. Considering the crucial role of epithelial-mesenchymal transition (EMT) in tumor progression and metastasis, we conducted Western blot analysis to evaluate the expression levels of E-Cadherin and N-Cadherin in A549 and H1299 cells after PSMB6 knockdown. The results showed that N-cadherin expression decreased while E-cadherin expression increased in A549 and H1299 cells with PSMB6 knockdown (*p* < 0.05, Figures 12E, F). This suggests that PSMB6 may promote the proliferation and migration of lung adenocarcinoma cells by regulating the EMT process.

## 4 Discussion

The proteasome is widely distributed in the cytoplasm and nucleus, exhibiting various protein hydrolase activities. It serves a pivotal function in governing various cellular processes, including the modulation of the cell cycle, regulation of apoptosis, transmission of signals, control of gene expression, and the process of translation. In malignant tumors, the proteasome has been acknowledged for its pivotal role in regulating anti-apoptotic pathways and proliferative signaling (Varga et al., 2017; Choi, 2001; Mani and Gelmann, 2005). The ubiquitin-proteasome system plays a significant role in cancer cell survival (i.e., evading apoptosis) and proliferation by targeting negative regulators of the cell cycle, such as p53 and p27, for degradation, as well as promoting the activation of NF-κB signaling (Shen et al., 2013). Furthermore, previous studies have reported successful implementation of proteasome inhibitors, including carfilzomib and bortezomib, in the clinical treatment of patients suffering from multiple myeloma (Chauhan et al., 2004; Chen et al., 2008).

PSMB6, responsible for encoding the β1 subunit of the proteasome, is crucial for the catalytic function and stable functioning of the proteasome. In this study, we identified a substantial elevation in the levels of PSMB6 within lung adenocarcinoma tissues, and this enhanced expression was linked to a diminished prognosis for the patients. These findings suggest that the high levels of PSMB6 expression may be inseparably linked to the degradation of negative regulators of the cell cycle, p53 and p27, as well as abnormal activation of the NF-κB signaling pathway. This could promote tumor cell growth and impact the anti-apoptotic pathways and proliferation signals of cancer cells, ultimately leading to poorer patient outcomes. Additionally, these results show that PSMB6 could serve as a prognostic marker and therapeutic target for malignant tumors, providing a new avenue for targeting the proteasome.

The tumor immune microenvironment (TIME) has long been acknowledged for its significant association with tumor recurrence, development, and metastasis (Fu et al., 2021). The impact of the TIME on patient survival has also been demonstrated in numerous cancer types (Li et al., 2017a). We observed that the low expression group of PSMB6 and the low-risk group showed higher immune cell infiltration and lower tumor purity. This indicates that PSMB6 may serve as a pivotal factor within the TIME by reducing immune cell infiltration, thereby aiding tumor cells in immune evasion. Indeed, research has shown that cytokines are pivotal in facilitating communication between cells within the tumor microenvironment (TME) and are strongly linked to the initiation, progression, and metastatic dissemination of tumors (Waldmann, 2018; Berraondo et al., 2019). Cytokines such as IFNγ, IFNα, and IL-2 promote anti-tumor responses in the TME, while dysregulation of cytokines produced by immune, malignant, and stromal cells are involved in the entire process of carcinogenesis and therapy response (Propper and Balkwill, 2022; Briukhovetska et al., 2021; Shalapour and Karin, 2019). Therefore, there is therapeutic potential in utilizing cytokines for their immunostimulatory effects and in neutralizing them when they are dysregulated. Our GO analysis revealed enrichment of immune-related functional pathways, indicating the involvement of cytokine production, leukocyte proliferation, and cellular activation in immune responses. This suggests that PSMB6 may alter the release of cytokines within the immune microenvironment by influencing leukocyte proliferation and cellular activation involved in immune responses, ultimately impacting the activity of lung adenocarcinoma cells. CD8^+^ T cells function as the key executors in the immune system’s fight against tumors, leveraging the display of peptides on the surface of cancer cells by Major Histocompatibility Complex (MHC) Class I molecules to recognize and eliminate the targeted cells (Bernal et al., 2012). In most instances, high-density memory T lymphocytes directed towards Th1 and robust CD8^+^ compartments are typically linked to improved prognostic outcomes (Becht et al., 2016b). Natural killer (NK) cells have the capacity to detect the reduction in Class I MHC expression and exert cytotoxicity that relies on direct contact. NK cells have been recognized as a prominent cellular population involved in facilitating immune responses against tumor cells (Vivier et al., 2012). We observed higher expression of CD8^+^ T cells and NK cells in the low-risk group with low PSMB6 expression. This suggests that PSMB6 may help promote the growth of lung adenocarcinoma cells by reducing the number of immune cells, such as CD8^+^ T cells and NK cells. Ultimately, this could lead to poorer prognosis for patients.

It is important to highlight that the KEGG analysis revealed a significant enrichment in MAPK pathways. The MAPK pathways encompass a three-tiered kinase module. The MAPK signaling pathway plays a role in various cellular processes such as cell proliferation, migration, differentiation, and apoptosis, regulating the biological functions of cells (Dhillon et al., 2007). Previous studies have indeed shown that the MAPK signaling pathway can influence the proliferation of lung cancer cells (Yu et al., 2022). This suggests a potential close association between PSMB6 and the MAPK pathway in promoting the growth and specialization of lung adenocarcinoma cells.

Interestingly, the GO analyses revealed significant enrichment of pathways associated with GTPase. Rho GTPases are pivotal regulators in numerous cellular activities, exerting their influence on cell proliferation, survival, and migration, among other processes. In addition to their roles in cellular processes, Rho GTPases participate in the interplay with the tumor microenvironment, where they modulate inflammation, which in turn can influence the progression of cancer (Crosas-Molist et al., 2022). Alterations in the expression of Rho GTPases or their upstream regulators have been frequently observed in cancer, with Rho GTPase often being overexpressed rather than downregulated (Haga and Ridley, 2016; Fritz et al., 2002; Del Pulgar et al., 2005). Rho GTPase signaling pathways are also of significant importance within immune cells (Bros et al., 2019). For example, RAC1P29S melanoma harbored increased PD-L1 expression compared to RAC1 wild type or other RAC1 mutants (Vu et al., 2015). In breast cancer, it has been observed that *in vitro*, the phosphorylation of moesin by ROCK can stabilize the levels of PD-L1. In mouse tumors, the systemic inhibition of ROCK hindered tumor progression by decreasing the expression of tumor-derived PD-L1, which in turn resulted in increased infiltration of CD4^+^ and CD8^+^ T cells (Meng et al., 2020). Additionally, inhibition of ROCK on tumors directly affected immune cell populations, enhancing dendritic cell-mediated phagocytosis of tumor cells, promoting antitumor immunity, and T-cell priming (Nam et al., 2018). In this study, we found that the high-risk group with elevated PSMB6 expression showed higher levels of PD-L1 expression. Additionally, in the low-risk group, immune scoring for immune cells such as CD8^+^ T cells was higher. Therefore, we hypothesize that Rho GTPase signaling in lung adenocarcinoma cells may serve as a key regulator of immune evasion. It achieves this by altering the expression level of PD-L1 and affecting the infiltration of immune cells, including CD8^+^ T cells. Ultimately, this promotes the growth and metastasis of LUAD cells.

Immune checkpoint molecules are a group of immunoregulatory receptors/ligands that play a significant role in maintaining the balance of the body’s immune response under normal physiological conditions. They influence the activation of T cells to prevent over-activation of the immune system (Morad et al., 2021; Wagner et al., 2021). One important strategy for tumors to escape immune surveillance is by regulating the expression of immune checkpoint molecules, thereby suppressing the anti-tumor response of the body (Khan et al., 2020). During the induction phase of the anti-cancer immune response, immune checkpoint molecules inhibit the activation of effector T cells by interfering with the interaction between CD80/CD86 and CD28. This weakening of the anti-tumor effects of T cells allows tumor cells to evade immune surveillance (Hosseini et al., 2020; Lee et al., 1998). We found that the expression of CD28, CD80, andCD86 was markedly reduced, suggesting that PSMB6 may decrease their expression to reduce the binding between CD80/CD86 and CD28, ultimately leading to immune evasion in lung adenocarcinoma cells. CD276 is a member of the immune checkpoint protein B7 family. There is evidence indicating that CD276 exhibits high expression in cancer cells as well as in activated immune cells infiltrating tumors. It assists tumor cells to evade surveillance by NK cells and cytotoxic T cells and is strongly linked to tumor cell proliferation and treatment resistance (Getu et al., 2023). In the high-risk group with elevated PSMB6 expression, there is a significant increase in CD276 expression. These results suggest that PSMB6 could potentially enhance the proliferation and metastasis of lung adenocarcinoma cells by upregulating CD276 expression and suppressing immune responses.

This study found that PSMB6 is relatively highly expressed in lung adenocarcinoma tissues and lung adenocarcinoma cells. *In vitro* experiments demonstrated that knocking down the expression of PSMB6 inhibited the proliferation, invasion, and metastasis of lung adenocarcinoma cells, while promoting their apoptosis. This indicates that PSMB6 is an important regulatory factor closely associated with the occurrence and development of lung adenocarcinoma. Epithelial-mesenchymal transition (EMT) plays a critical role in the proliferation and migration of tumor cells (Li et al., 2017b; Tsutsumi et al., 2017). Through EMT, tumor cells can alter the expression of surface antigens, thereby reducing the likelihood of recognition by immune cells. Additionally, the cytokines and chemokines secreted by tumor cells during the EMT process can recruit immunosuppressive cells, further inhibiting the immune response. These mechanisms enable tumor cells to possess a stronger escape ability under immune surveillance (Chaffer and Weinberg, 2011). Recent studies have shown that the proteasome is closely related to EMT in the development of gastric cancer (Wang et al., 2023), but the interaction between PSMB6 and EMT in lung adenocarcinoma has not been reported. Therefore, we used Western blot experiments to detect the expression of E-cadherin and N-cadherin (EMT markers) in lung adenocarcinoma cells. The results indicated that knocking down PSMB6 expression increased E-cadherin expression while inhibiting N-cadherin expression. Combined with the observation that the PSMB6 high expression group had a lower immune score, we speculate that PSMB6 may influence the immune microenvironment through the EMT pathway, thereby promoting the proliferation and invasion of lung adenocarcinoma.

Our study is not without limitations. First, we utilized a substantial amount of data from online databases such as GEO and TCGA. The patients in these public databases exhibit heterogeneity in clinical characteristics, including variations in immunotherapy and staging, which may impact the accuracy of the proposed predictive models. Therefore, it is necessary to use larger prospective cohorts to further validate these risk prediction models. Second, conducting animal experiments to elucidate the role of PSMB6 in LUAD, particularly its impact on the tumor immune microenvironment, is essential.

## Data Availability

The raw data supporting the conclusions of this article will be made available by the authors, without undue reservation.
