# Corrigendum: Immune regulation and prognostic prediction model establishment and validation of PSMB6 in lung adenocarcinoma

**DOI:** 10.3389/fgene.2024.1531321

**Published:** 2024-12-04

**Authors:** Haiyang Zhao, Kexin Luo, Meihan Liu, Yuanze Cai, Siman Liu, Shijuan Li, Yongsheng Zhao, Hongpan Zhang

**Affiliations:** ^1^ Affiliated Hospital of North Sichuan Medical College, Nanchong, Sichuan, China; ^2^ North Sichuan Medical College, Nanchong, China; ^3^ Department of Thoracic Surgery, Affiliated Hospital of North Sichuan Medical College, Nanchong, Sichuan, China; ^4^ North Sichuan Medical College, Innovation Centre for Science and Technology, Nanchong, China; ^5^ Department of Oncology, Affiliated Hospital of North Sichuan Medical College, Nanchong, China; ^6^ Nanchong Central Hospital, Nanchong, Sichuan, China; ^7^ Therapeutic Proteins Key Laboratory of Sichuan Province, Nanchong, China

**Keywords:** lung adenocarcinoma, PSMB6, immunotherapy, immune infiltration, tumor immune microenvironment

In the published article, there was an error regarding the **Affiliations** listed for Haiyang Zhao, Kexin Luo, Meihan Liu, Yongsheng Zhao and Hongpan Zhang. In addition to their existing **Affiliations**, they should also have Affiliated Hospital of North Sichuan Medical College, Nanchong, Sichuan, China listed to their names.

The corrected **Affiliations** are listed below:

Haiyang Zhao^1,2,3,4,†^, Kexin Luo^1,2,3,4,†^, Meihan Liu^1,2,4,5,†^, Yuanze Cai^2^, Siman Liu^2^, Shijuan Li^6^, Yongsheng Zhao^1,2,3,^*, Hongpan Zhang^1,2,5,7,^*

1.Affiliated Hospital of North Sichuan Medical College, Nanchong, Sichuan, China

2.North Sichuan Medical College, Nanchong, China

3.Department of Thoracic Surgery, Affiliated Hospital of North Sichuan Medical College, Nanchong, Sichuan, China

4.North Sichuan Medical College, Innovation Centre for Science and Technology, Nanchong, China

5.Department of Oncology, Affiliated Hospital of North Sichuan Medical College, Nanchong, China

6.Nanchong Central Hospital, Nanchong, Sichuan, China

7.Therapeutic Proteins Key Laboratory of Sichuan Province, Nanchong, China

The authors apologize for this error and state that this does not change the scientific conclusions of the article in any way. The original article has been updated.

